# Lanatoside C, a Novel Senolytic, Ameliorates Atherosclerosis in Mice

**DOI:** 10.14336/AD.2025.1219

**Published:** 2026-04-21

**Authors:** Eok-Cheon Kim, Youlim Son, Seon-Hui Kim, Soo-Ji Kim, So-Young Park, Jae-Ryong Kim

**Affiliations:** ^1^Department of Biochemistry and Molecular Biology, Daegu 42415, Republic of Korea; ^2^Senotherapy-based Metabolic Disease Control Research Center, Daegu 42415, Republic of Korea; ^3^Department of Physiology, College of Medicine, Yeungnam University, Daegu 42415, Republic of Korea

**Keywords:** lanatoside C, senolytic, atherosclerosis, age-related disease

## Abstract

Cellular senescence, a state of irreversible cell cycle arrest, contributes to aging and age-related diseases. Senolytics targeting cellular senescence could be applied to the prevention and treatment of age-related diseases. In this study, we identified lanatoside C (Lana C) as a senolytic compound. Lana C, a cardiac glycoside used for the treatment of cardiovascular diseases, is known to inhibit the transmembrane protein sodium-potassium adenosine triphosphatase (Na^+^/K^+^-ATPase). We found that Lana C depolarized and acidified senescent human umbilical vein endothelial cells (HUVECs), making them susceptible to apoptosis. The senolytic activity of Lana C was inhibited by potassium chloride (KCl) and Z-VAD-FMK (ZVF), a widely used pan-caspase inhibitor. Additionally, Lana C significantly ameliorated the senescence burden and the formation of atherosclerotic lesions in apolipoprotein E (*ApoE^-/-^*) or low-density lipoprotein receptor (*Ldlr^-/-^*) knockout mice. These results suggest that Lana C could be a promising senolytic for age-related diseases.

## INTRODUCTION

Senescence is a natural process that occurs in the body, and the accumulation of senescent cells (SCs) with age is thought to contribute to aging and age-related diseases [1-4]. SCs release a myriad of pro-inflammatory factors and bioactive molecules. The term senescence-associated secretory phenotype (SASP) defines the ability of the SCs to express and secrete these factors which can induce chronic inflammation and tissue dysfunction, and promote the development of age-related pathologies, including atherosclerosis, fibrosis, and metabolic dysfunction.

In recent years, the development of senotherapeutics—senolytics that selectively eliminate SCs and senomorphics that attenuate the pathological SASP to cause senostasis—has garnered significant attention as a promising strategy to combat age-related diseases and extend the healthspan [5-7]. Senotherapeutic compounds have demonstrated the potential to improve tissue regeneration and restore tissue homeostasis by efficiently clearing SCs, thereby alleviating the detrimental effects of the SASP. Various compounds have been investigated as potential senolytics, including dasatinib, quercetin, fisetin, ABT-263, and senomorphics, including rapamycin, metformin, resveratrol, and aspirin, among others [8-11]. These compounds work through different mechanisms to induce the death of SCs, such as disrupting anti-apoptotic pathways or interfering with SC survival signals, and suppress the SASP components through the regulation of the mammalian target of rapamycin (mTOR), nuclear factor-kappa B (NF-κB), phosphor-inositide-3-kinase–protein kinase B/Akt (PI3K/Akt), and Janus kinase/signal transducers and activators of transcription (JAK/STAT) pathways [8]. As research in the field of senotherapeutic advances, novel compounds with high senotherapeutic potency and safety profiles are being actively sought.

In an attempt to explore novel senotherapeutics by drug repositioning, we screened 2,150 FDA-approved clinical trial compounds obtained from the Korea Chemical Bank and discovered that lanatoside C (Lana C) might have a senolytic activity. Lana C is a cardiac glycoside, a class of compounds derived from plants, known for their therapeutic effects on the heart. It is extracted from the plant *Digitalis lanata*, commonly known as the woolly foxglove or Grecian foxglove [12]. Cardiac glycosides have been used for many years in the treatment of various heart conditions, particularly congestive heart failure and certain types of arrhythmias [13-15]. These compounds exert their pharmacological effects by inhibiting the Na^+^/K^+^-ATPase pump, which leads to an increase in the intracellular calcium levels in cardiac muscle cells [16-18]. This, in turn, enhances the contractile force of the heart, improves cardiac output, and helps alleviate the symptoms associated with heart failure [13, 18, 19]. In addition, several cardiac glycosides (ouabain, proscillaridin, and digoxin) have been reported to be senolytic compounds that could potentially be used to develop novel treatments against age-related diseases [20, 21]. However, the effects of Lana C on cellular senescence and age-related diseases remain unidentified.

In this study, we tried to explore the senolytic effects of Lana C and its potential in mitigating age-related diseases. Through *in vitro* and *in vivo* experiments, we elucidated the underlying mechanisms of the senolytic action of Lana C in human primary cells as well as its therapeutic efficacy against senescence-related pathologies such as atherosclerotic plaque formation in murine models.

## MATERIALS AND METHODS

### Materials

Human umbilical vein endothelial cells (HUVECs) and endothelial cell basal medium-2 (EBM-2) containing several growth factors were purchased from Lonza Inc. (Walkersville, MD, USA). The Cell Counting Kit-8 (CCK-8) and lactate dehydrogenase (LDH) assay kit were obtained from Dojindo Molecular Technologies (Rockville, MD, USA). 5-bromo-4-chloro-3-indolyl-β-D-galactopyranoside (X-gal) was purchased from Amresco LLC (Solon, Ohio, USA). Doxorubicin was purchased from Ildong Pharmaceutical Co. Ltd. (Seoul, Republic of Korea). Lanatoside C was obtained from Sigma-Aldrich (St. Louis, MO, USA). Amiloride, dasatinib, and quercetin were purchased from Tocris (Avonmouth, Bristol, UK). ABT-263 (navitoclax) was acquired from Selleckchem (Houston, TX, USA). Z-VAD-FMK was purchased from MedChemExpress (Monmouth Junction, NJ, USA). Rapamycin was purchased from the Tokyo Chemical Industry (Kita-ku, Tokyo, Japan). The primary antibodies used in this study for western blot analysis are listed in Supplementary Table 1.

### Preparation of prematurely senescent (PS) cells

HUVECs were seeded at 1×10^5^ cells in 60 mm culture dishes and incubated overnight at 37°C in a 5% CO_2_ humidified incubator. Cells were then washed twice with DMEM containing 1% antibiotics and then treated with 0.5 μM doxorubicin for 4 h. After washing, cells were further incubated in the culture media for 4 days.

### Cell culture and preparation of replicatively senescent (RS) cells

HUVECs were maintained in EBM-2 containing several growth factors. Cells were seeded at 1 × 10^5^ cells per 100 mm culture dish and incubated at 37°C in a 5% CO_2_ humidified incubator. When subcultures reached 80-90% confluence, serial passaging was performed by trypsinization, and the population doubling time (PDT) was monitored for further experiments. PDT was calculated using the geometric equation: PDT = (Tf-Ti)/(log_2_F/log_2_I), where Tf is the final incubation time, Ti is the initial incubation time, F is the final population number, and I is the initial population number. For experiments, young HUVECs (PDT < 2 days) and replicatively senescent HUVECs (PDT > 15 days) were used.

### Cell Counting Kit-8 (CCK-8) analysis

The CCK-8 reagent was added to each well at a concentration of 10 μl/100 μl media/well and incubated for 2 h in an incubator. Absorbance was measured at 450 nm using a spectrophotometer. The cell survival rate was expressed as a relative value with the absorbance of the control group treated only with dimethylsulfoxide (DMSO) set at 100%.

### Cell viability assay

HUVECs were seeded in 24-well plates (3,000 cells/well for young cells and 10,000 cells/well for PS or RS cells) and treated with the indicated concentrations of Lana C for 4 days. Subsequently, cell viability was measured using the cell viability assay kit (#EZ-3000, DoGenBio Co., Seoul, Republic of Korea), or by counting cells in 5 random fields after staining with eosin.

### Lactate dehydrogenase (LDH) assay

LDH activity was measured in media using an LDH assay kit (Dojindo Molecular Technologies, Rockville, MD, USA).

### Senescence associated β-galactosidase (SAβG) staining of cells

HUVECs was washed twice with PBS and fixed with 3.7% (v/v) formaldehyde in PBS for 3 min at room temperature at the end of the experiment. Cells were then incubated in an SAβG staining solution consisting of 1 mg/ml 5-bromo-4-chloro-3-indolyl-β-D-galactoside, 40 mM sodium citrate-phosphate (pH 5.8), 5 mM potassium ferricyanide, 5 mM potassium ferrocyanide, 150 mM NaCl and 2 mM MgCl_2_ for 18 h at 37°C. Finally, the cells were washed twice with PBS, stained with 0.5% eosin solution for 3 min, and the percentage of SAβG-stained positive blue cells observed under an optical microscope was calculated.

### Membrane potential, intracellular Ca^2+^, and H^+^ measurement

Cell membrane potential, intracellular Ca^2+^ and H^+^ were measured according to the method provided by the company (Thermo Fisher Scientific and AAT Bioquest). Briefly, cells were seeded in a confocal plate, treated with 250 nM Bis-(1,3-dibutylbarbituric acid) trimethine oxonol (DiBAC4(3)), excitation 488 nm, emission 510 nm, Thermo Fisher Scientific, Eugene, OR, USA), 5 μM Cal-520®, AM (excitation 490 nm, emission 525 nm, AAT Bioquest, Pleasanton, CA) and 1× pHrodo™ Green AM Ester staining solution (excitation 509 nm, emission 533 nm, Thermo Fisher Scientific, Eugene, OR) for 30 min, treated with Lana C, and the changed membrane potential, intracellular Ca^2+^ and H^+^ was observed using a K1-Fluo confocal laser microscope (Nanoscope Systems Inc. Daejeon, Republic of Korea). The relative fluorescence intensity (RFI) was expressed as a ratio of the fluorescence intensity after the Lana C treatment and the fluorescence intensity before the Lana C treatment.

### Animal models

*Apolipoprotein E* deficient (*ApoE^-/-^*) mice and low-density lipoprotein receptor deficient (*Ldlr^-/-^*) mice were kindly donated by G. T. Oh (Ewha Womans University, Seoul, Republic of Korea), luciferase knockin mouse (*p16-luc*) mice were kindly provided by Eiji Hara (Osaka University, Japan). Animals were housed in a light- and temperature-controlled room and had free access to drinking water and a standard rodent diet. All animal studies were approved by the Institutional Animal Care and Use Committee of the College of Medicine, Yeungnam University (YUMC-AEC2021-022, YUMC-AEC2021-025).

### Animal experiments and treatment with Lanatoside C (Lana C)

To examine the effect of Lana C on senescence of the lung tissues *in vivo*, 80–100-week-old male *p16-luc* mice were administered the vehicle or Lana C intraperitoneally twice a week for 8 weeks (Veh, n=3; Lana C, n= 4). Lana C was dissolved in DMSO and diluted in PBS and injected at 0.985 mg/kg body weight. To evaluate the effects of Lana C on atherosclerotic lesion formation in mice, *ApoE^-/-^* and *Ldlr^-/-^* mice were fed a purified diet to match Paigen's Atherogenic Rodent Diet (Research Diets Inc., New Brunswick, NJ) for 6 weeks. In the case of female *Ldlr^-/-^* mice, they were fed high fat diet for 8 weeks. The sex, age, and number of the *ApoE^-/-^* and *Ldlr^-/-^* mice used are shown in Supplementary Table 2. Next, the mice were administered the vehicle or Lana C (0.985 mg/kg) intraperitoneally twice a week for 6 weeks. The *ApoE^-/-^* and *Ldlr^-/-^* mice were sacrificed and their aortas were harvested and analyzed. The food intake and body weight of all mice were measured once a week.

### Bioluminescence imaging of animals

Bioluminescence imaging was performed using the SPECTRAL Ami/AmiX system (Spectral Instruments Imaging, LLC, Tucson, AZ) for the detection of *in vivo* luciferase activity. The *p16-luc* mouse was anesthetized and hair removal cream was applied to remove the fur. D-luciferin (3.3 mg in 100 μl PBS) was injected intraperitoneally 5 min before imaging. Bioluminescence intensity was obtained for a 5 min exposure.

### Oil Red O (ORO) staining for quantification of the atherosclerotic area in mouse aortas

Adventitial fat was removed from the aortas of the *ApoE^-/-^* and *Ldlr^-/-^* mice and the aortas were fixed in a neutral buffered formalin solution overnight. After fixation, the aortas were washed with PBS and dehydrated with propylene glycol at room temperature. Aortas were then incubated in an ORO solution at room temperature. The aortas were washed with a solution of propylene glycol and PBS and fixed on a silicone rubber plate with microneedles. Finally, images of the aorta were taken using a light microscope, and the percentage of ORO positive area was measured with the Image J software.

### SAβG staining of tissues

Mice treated with the vehicle or Lana C were anesthetized and perfused with PBS. Subsequently, the aortas from the *ApoE^-/-^* and *Ldlr^-/-^* mice were excised. The tissues were fixed with 3.7% (v/v) formaldehyde in PBS at room temperature for 30 min. After washing with PBS, the tissues were incubated for 5 h at 37°C in an SAβG staining solution. The percentages of SAβG-stained positive area relative to the whole tissues were measured with the Image J software.

### Protein extraction

Cells were washed with ice-cold PBS and harvested by scraping in 50 μl of ice-cold radioimmunoprecipitation assay (RIPA) buffer (25 mM Tris-HCl, pH 7.4, 150 mM KCl, 5 mM NaF, and 1 mM PMSF). The cells were ruptured by vortexing twice for 30 sec at 30 min intervals on ice and centrifuged at 13,000 rpm for 15 min at 4°C. The aorta tissues were homogenized in 5 volumes of an ice-cold RIPA buffer and centrifuged at 13,000 rpm for 15 min at 4°C. Protein concentrations in the supernatants were quantified by the bicinchoninic acid method (Pierce Biotechnology Inc., Rockford, IL) using bovine serum albumin as a standard.

### Western blot analysis

Proteins were separated by 10% or 12% sodium dodecyl-sulfate-polyacrylamide gel electrophoresis (SDS-PAGE) and then transferred to nitrocellulose membranes. Next, the membrane was blocked in 1 × TTBS (10 mM Tris-HCl pH 7.5, 150 mM NaCl, and 0.1% Tween-20) containing 5% skim milk for 45 min at room temperature. The membrane was incubated overnight in a 4°C chamber with primary antibodies (Supplementary Table 1). A secondary antibody (1:3000) was then applied to the membrane for 60 min at room temperature. After washing the membrane three times with 1 × TTBS for 45 min, antigen-antibody complexes were detected using a Western blotting luminol reagent (Santa Cruz Biotech Inc., Santa Cruz, CA). Proteins were visualized using a LAS-3000 imaging system (Fuji Film Corp., Stamford, CT). Glyceraldehyde-3-phosphate dehydrogenase (GAPDH) and actin were used as controls for protein loading. The intensity of the protein band relative to each GAPDH and actin signal was determined using the Image J program and normalized to 1.0 relative to the control cells by taking the average of the results of the three or four separate experiments.

### Statistical analysis

The results are presented as means ± SEM. Statistical analysis was performed using GraphPad Prism 8. Data normality was assessed using the D'Agostino-Pearson or Kolmogorov-Smirnov test. For normally distributed data, statistical significance was evaluated using one-way or two-way ANOVA with an appropriate post-hoc test, or a two-tailed Student’s *t* test. If the data were not normally distributed or if the sample size was too small (N < 6), the Mann-Whitney U test was applied. Differences were deemed significant at *p* < 0.05 (*) and *p* < 0.01 (**).

## RESULTS

### Elucidation of the senolytic activity of Lana C

#### Screening of senotherapeutics: Identification of Lana C

We attempted to identify senotherapeutic candidates from 2,150 FDA-approved clinical compounds by studying them in human primary cells and identified Lana C as a senolytic agent. To summarize, we initially treated PS cells with the compounds and then selected the compounds that killed SCs or ameliorated the senescence phenotypes of the PS cells. Subsequently, we treated the RS cells and young cells with the selected compounds and finally identified several senotherapeutic candidates with relevant activity, including Lana C, which is a cardiac glycoside. Since digoxin, a cardiac glycoside has been previously reported to have senolytic activity [20], we sought to investigate whether Lana C would also exhibit senolytic activity in *in vitro* as well as *in vivo* animal models.

#### Results of cell counting and cell viability assays

**Figure 1.**
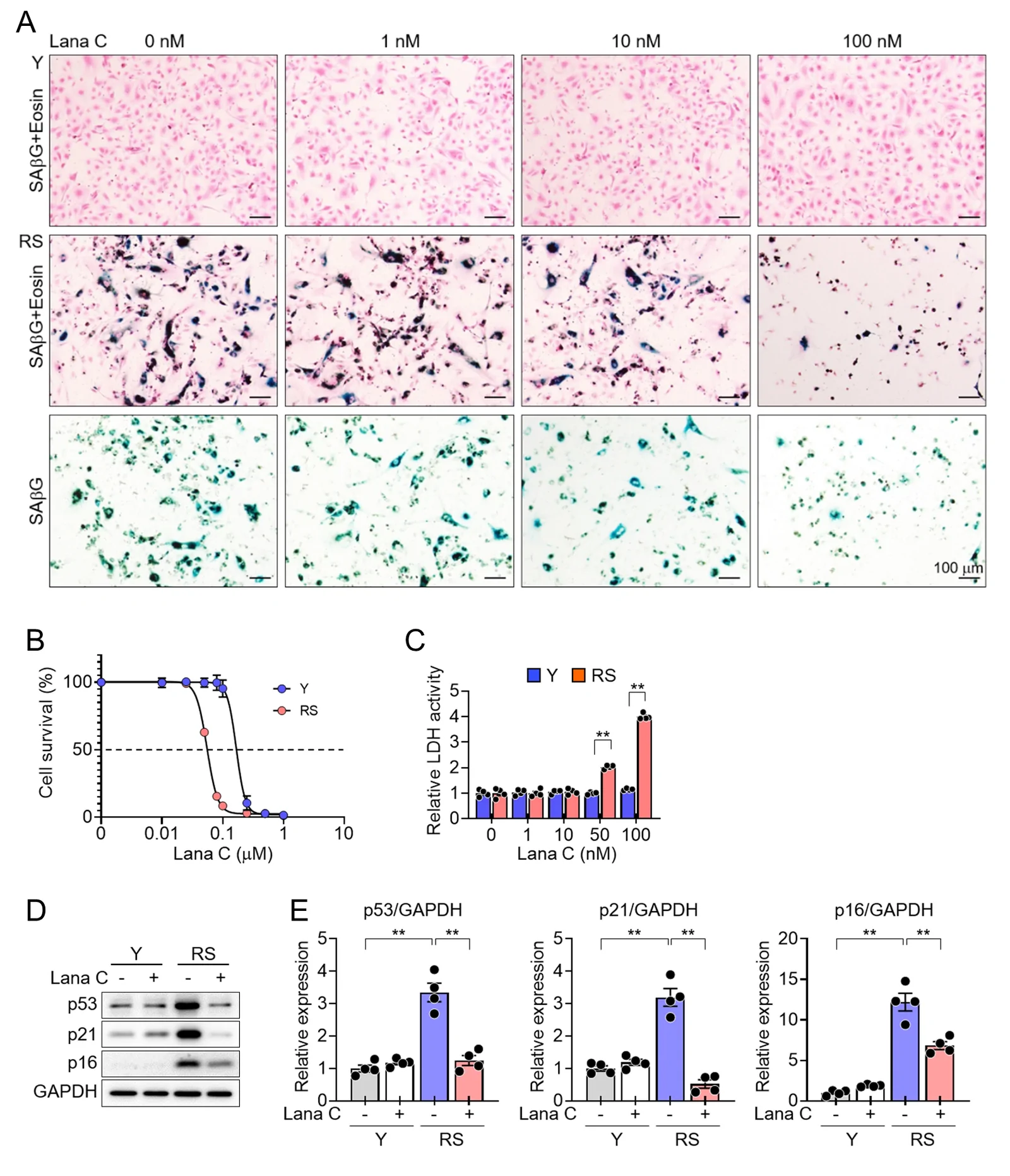
**Identification of the in vitro senolytic activity of Lana C.** Young or RS HUVECs were treated with the indicated concentrations of Lana C for 4 days. **(A)** Representative cell images from 4 independent experiments (n=4 in each group). **(B)** Cell viability measured by cell counting kit-8 (n=4 in each group). **(C)** LDH activity in culture media (n=4 in each group). **(D)** Western blotting images. **(E)** Quantification of the levels of p53, p21^Cip1^, and p16^Ink4a^ proteins (n=4 in each group). Values are presented as means ± SEM and data were analyzed with two-way ANOVA followed by a post-hoc test. **p<0.01. Abbreviations: Y, young; RS, replicatively senescent; HUVEC, human umbilical vein endothelial cell; Lana C, lanatoside C; LDH, lactate dehydrogenase; SAβG, senescence associated β-galactosidase.

We confirmed that the treatment with Lana C induced cell death in PS and RS HUVECs in a dose-dependent manner, and the cell death induced by Lana C was greater in senescent HUVECs than in young HUVECs (Figs. 1A and B, and Supplementary Figs. 1A and B), as confirmed by cell counting. We further tested whether the cytotoxicity of Lana C is selective to senescent HUVECs by measuring LDH activity in the culture media. LDH is a stable cytoplasmic protein in all types of cells and is released into the culture medium through damaged plasma membranes [22]. Lana C treatment enhanced LDH activity in a dose-dependent manner in RS HUVECs but not in young HUVECs (Fig. 1C), suggesting that Lana C might have senolytic activity in HUVECs. In addition, Lana C treatment reduced the levels of senescence marker proteins, including p53, p21^Cip1^, and p16^Ink4a^ [23], which were elevated in senescent cells (Figs. 1D and E, and Supplementary Figs. 1C and D).

#### Senolytic activity of Lana C in transgenic p16-luc mice

**Figure 2.**
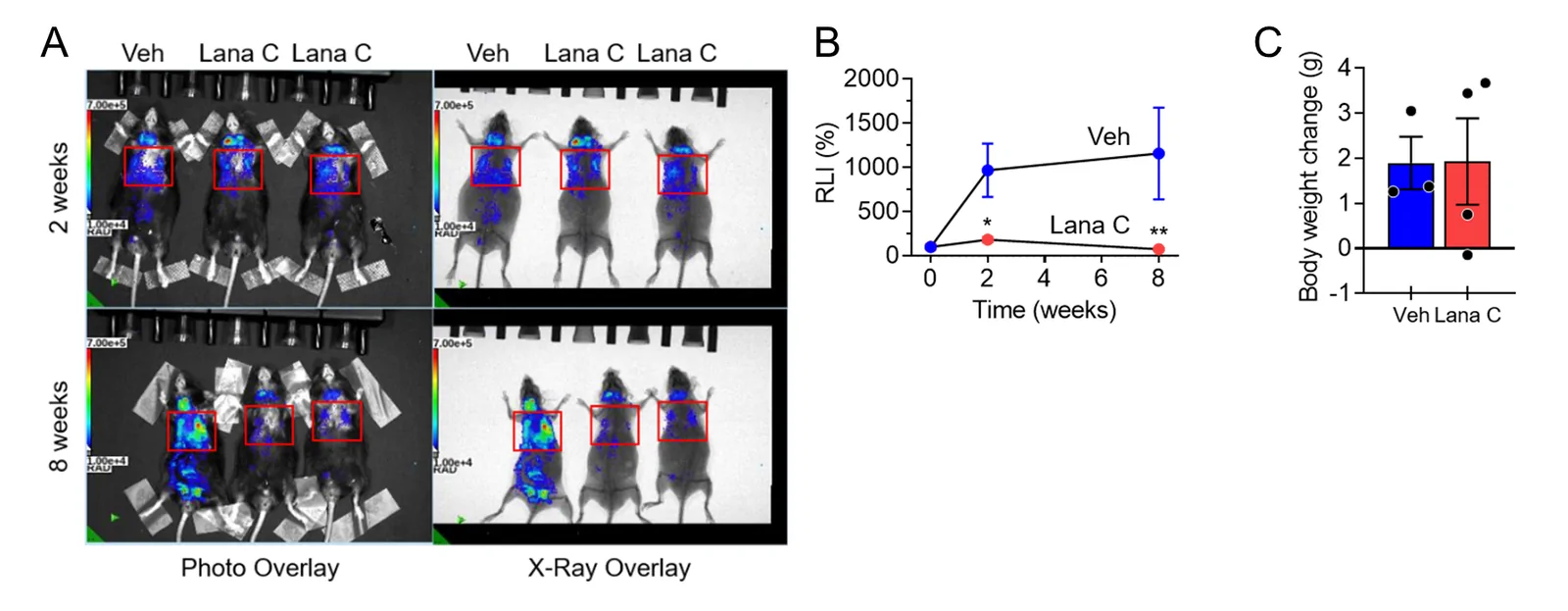
**Identification of the *in vivo* senolytic activity of Lana C.** *p16-luc* mice were treated with the vehicle or Lana C for 8 weeks and bioluminescence was measured. **(A)** Representative luminescence images of *p16-luc* mice treated with the vehicle or Lana C. **(B)** Quantification of relative luminescence intensity (RLI) (Veh, n=3; Lana C, n=4). **(C)** Body weight change of *p16-luc* mice (Veh, n=3; Lana C, n=4). Values are presented as means ± SEM and data were analyzed with two-way ANOVA followed by a post-hoc test. *p<0.05, **p<0.01. Abbreviations: Veh, vehicle; Lana C, lanatoside C.

We further investigated whether the *in vitro* senolytic activity of Lana C is exerted *in vivo* in animal tissues. To confirm the senolytic activity of Lana C *in vivo*, its effects were tested on the ablation of SCs in transgenic *p16-luc* mice harboring the entire human p16^Ink4a^ locus tagged with firefly luciferase and reported increasing bioluminescence concurrently with p16^Ink4a^ [24]. p16^Ink4a^, a key molecule in cellular senescence, is upregulated with age in various animal tissues [25]. When *p16-luc* mice (80-100 weeks of age) were treated with Lana C or the vehicle twice a week for 8 weeks, the Lana C administration significantly reduced lung bioluminescence compared with the vehicle treatment without body weight changes (Fig. 2). These results indicate that SCs that were accumulated with age in the lung tissues of *p16-luc* mice were removed by the Lana C treatment, demonstrating the *in vivo* senolytic activity of Lana C.

### Lanatoside C inhibits vascular senescence and atherosclerotic plaque formation in HFD-fed *ApoE^-/-^* and *Ldlr^-/-^* mice

**Figure 3.**
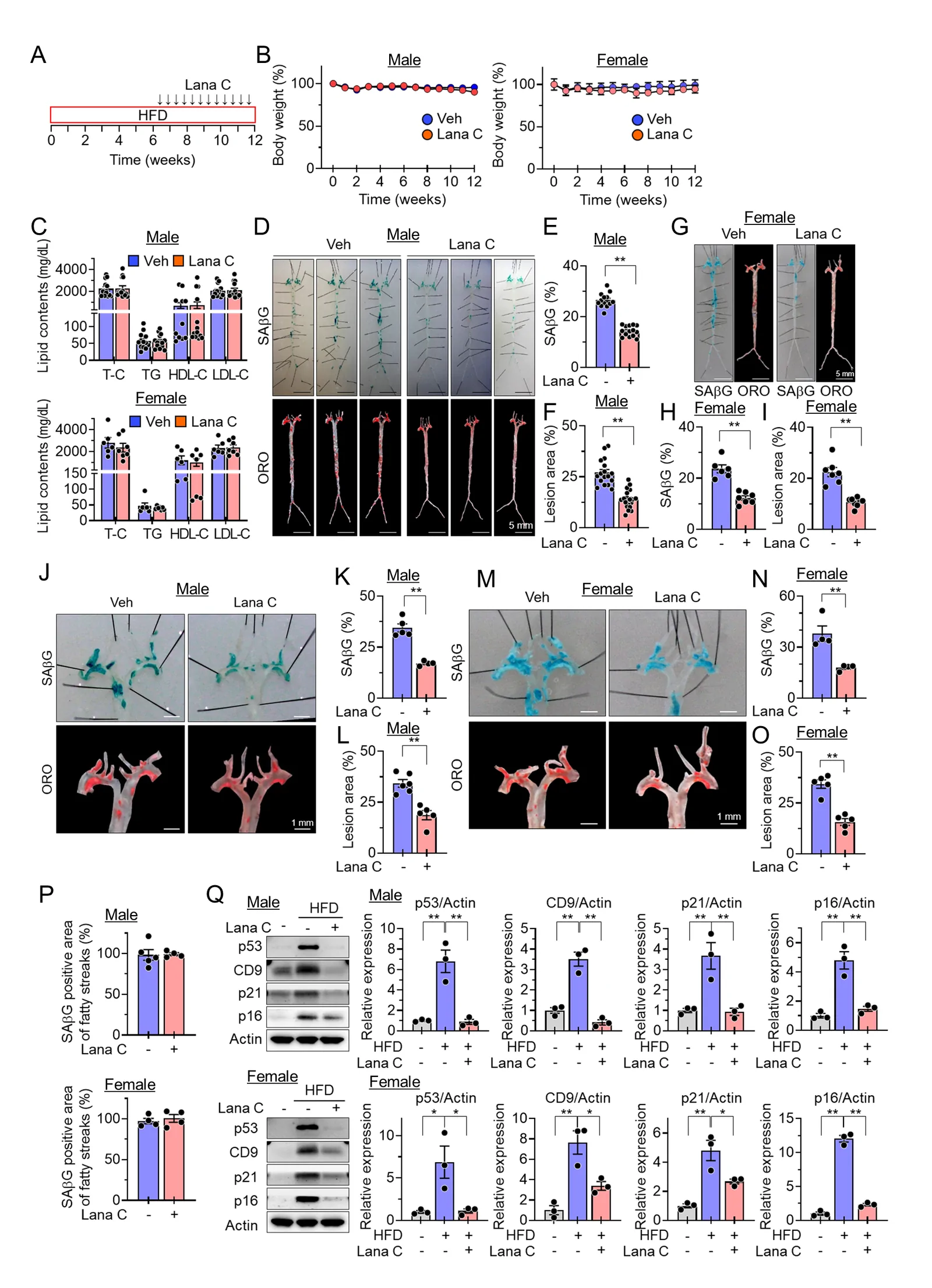
**Lana C reduces the senescence burden and the formation of atherosclerotic lesions in HFD-fed male and female *ApoE^-/-^* mice.** Male and female *ApoE^-/-^* mice were first fed a high-fat diet for 6 weeks and then administered Lana C or vehicle intraperitoneally twice a week for an additional 6 weeks. **(A)** Experimental scheme. **(B)** Body weight changes in male (Veh, n=13; Lana C, n=14) and female (Veh, n=6; Lana C, n=7) mice. **(C)** Serum lipid contents in male (Veh, n=13; Lana C, n=14) and female (Veh, n=6; Lana C, n=7) mice. **(D)** Representative *en face* aortic images stained with SAβG and ORO in male mice. **(E)** Percentages of SAβG positive areas in *en face* aorta of male mice (Veh, n=13; Lana C, n=14). **(F)** Quantification of atherosclerotic lesion areas in *en face* aorta of male mice (Veh, n=17; Lana C, n=19). **(G)** Representative *en face* aortic images stained with SAβG and ORO in female mice. **(H)** Percentages of SAβG positive areas in *en face* aorta of female mice (Veh, n=6; Lana C, n=7). **(I)** Quantification of atherosclerotic lesion areas in *en face* aorta of female mice (Veh, n=7; Lana C, n=8). **(J)** Representative *en face* aortic arch images stained with SAβG and ORO staining in male mice. **(K)** Percentages of SAβG positive areas in *en face* aortic arches in male mice (Veh, n=5; Lana C, n=4). **(L)** Quantification of the atherosclerotic lesion areas in *en face* aortic arches in male mice (Veh, n=6; Lana C, n=5). **(M)** Representative *en face* aortic arch images stained with SAβG and ORO in female mice. **(N)** Percentages of SAβG positive areas in *en face* aortic arches in female mice (Veh, n=4; Lana C, n=4). **(O)** Quantification of the atherosclerotic lesion areas in *en face* aortic arches in female mice (Veh, n=5; Lana C, n=5). **(P)** Percentages of SAβG positive areas of fatty streaks in male (Veh, n=5; Lana C, n=4) and female (Veh, n=4; Lana C, n=4) mice. **(Q)** Representative Western blotting images and quantification of the levels of p53, CD9, p21^Cip1^, and p16^Ink4a^ proteins (n=3 in each group). Values are presented as means ± SEM and data were analyzed with two-tailed Student’s *t* test or one-way ANOVA followed by a post-hoc test. *p<0.05, **p<0.01. Abbreviations: Lana C, lanatoside C; HFD, high-fat diet; Veh, vehicle; ORO, Oil Red O; SAβG, senescence associated β-galactosidase; T-C, total cholesterol; TG, triglyceride; HDL-C, high density lipoprotein cholesterol; LDL-C, low density lipoprotein cholesterol.

Atherosclerosis is an age-related chronic, complex, and progressive disease characterized by the accumulation of plaque within the arteries, and is an important cause of morbidity and mortality in industrialized countries [26]. Accumulating evidence suggests that removing SCs inhibits arteriosclerotic plaque formation. Therefore, we sought to investigate the senolytic effect of Lana C on atherosclerotic plaque formation in *ApoE^-/-^* or *Ldlr^-/-^* mice. The *ApoE^-/-^* mice were first fed a high-cholesterol diet for 6 weeks and then administered the vehicle or Lana C (0.985 mg/kg in PBS) intraperitoneally twice a week for an additional 6 weeks (Fig. 3A). There were no differences in body weight or blood lipid profiles between the Lana C-treated and vehicle-treated groups in both male and female mice (Figs. 3B and C). Lana C administration resulted in a reduction in the SAβG positive areas and the formation of atherosclerotic lesions in *en face* aortas (Figs. 3D-I) and *en face* aortic arches (Figs. 3J-O) in the *ApoE^-/-^* mice compared with the vehicle group. The SAβG positive areas were almost identical to the ORO-stained fatty streak portions of the aortas (Fig. 3P). In addition, the senescence marker proteins, such as p53, CD9, p21^Cip1^, and p16^Ink4a^ in the aortic tissues, which are upregulated by the HFD, were also decreased by the Lana C treatment (Fig. 3Q).

**Figure 4.**
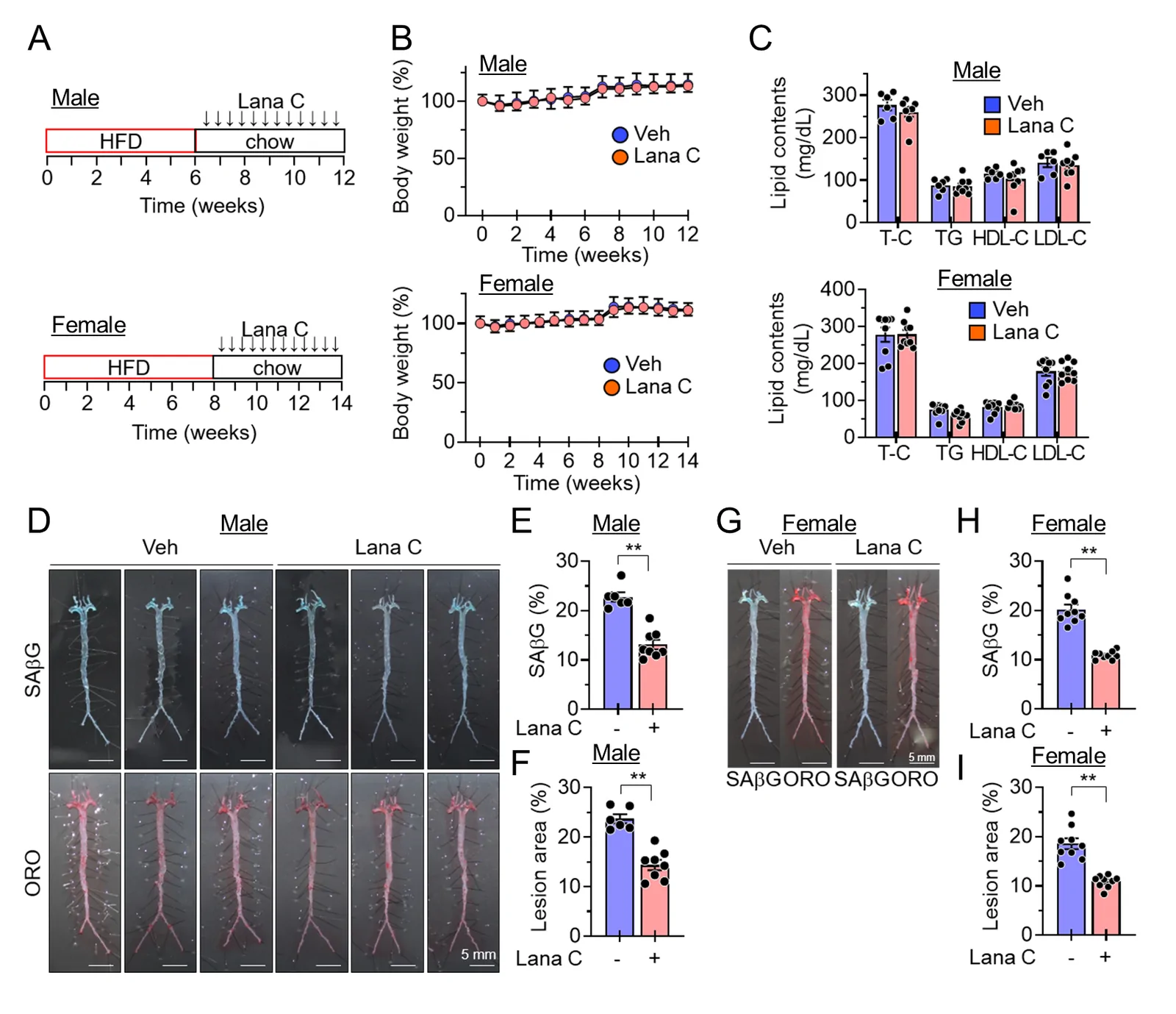
**Lana C mitigates the senescence burden and the formation of atherosclerotic lesions in HFD-fed *Ldlr^-/-^* mice.** *Ldlr^-/-^* mice were fed a high-cholesterol diet for 6 weeks (male) or 8 weeks (female) and then changed to a normal chow diet and administered Lana C or vehicle intraperitoneally twice a week. **(A)** Experimental scheme. **(B)** Body weight changes in male (Veh, n=6; Lana C, n=8) and female (n=9 per group) mice. **(C)** Serum lipid contents in male (Veh, n=6; Lana C, n=8) and female (n=9 per group) mice. **(D)** Representative *en face* aorta images stained with SAβG and ORO images in male mice. **(E)** Percentages of SAβG positive areas in *en face* aorta in male mice (Veh, n=6; Lana C, n=8). **(F)** Quantification of atherosclerotic lesion areas in *en face* aorta in male mice (Veh, n=6; Lana C, n=8). **(G)** Representative *en face* aorta images stained with SAβG and ORO in female mice. **(H)** Percentages of SAβG positive areas in *en face* aorta in male mice (n=9 in each group). **(I)** Quantification of atherosclerotic lesion areas in *en face* aorta in female mice (n=9 in each group). Values are presented as means ± SEM and data were analyzed with two-tailed Student’s *t* test. **p<0.01. Abbreviations: Lana C, lanatoside C; Veh, vehicle; ORO, Oil Red O; HFD, high-fat diet; T-C, total cholesterol; TG, triglyceride; HDL-C, high density lipoprotein cholesterol; LDL-C, low density lipoprotein cholesterol; SAβG, senescence associated β-galactosidase.

Next, the senolytic effects of Lana C were analyzed using *Ldlr^-/-^* mice, another well-established animal model for atherosclerosis. *ApoE^-/-^* mice are often preferred for studies of early lesion development, while *Ldlr^-/-^* mice may be used when studying the progression and management of established atherosclerotic plaques. *Ldlr^-/-^* mice were fed a high-cholesterol diet for 6 weeks in males and 8 weeks in females and then fed a normal chow diet and administered Lana C or the vehicle intraperitoneally twice a week for an additional 6 weeks (Fig. 4A). No differences in body weight and blood lipid contents due to Lana C administration were observed in both male and female mice (Figs. 4B and C). Lana C administration resulted in a reduction in the SAβG positive areas and the formation of atherosclerotic lesions in aortic *en face* in the *Ldlr^-/-^* mice compared to the vehicle group (Figs. 4D-I). These results suggest that Lana C ameliorates atherosclerotic plaque formation in mice.

### Senolytic activity of Lana C mediated through selective membrane depolarization and detrimental acidification induced by Na^+^/K^+^-ATPase inhibition

**Figure 5.**
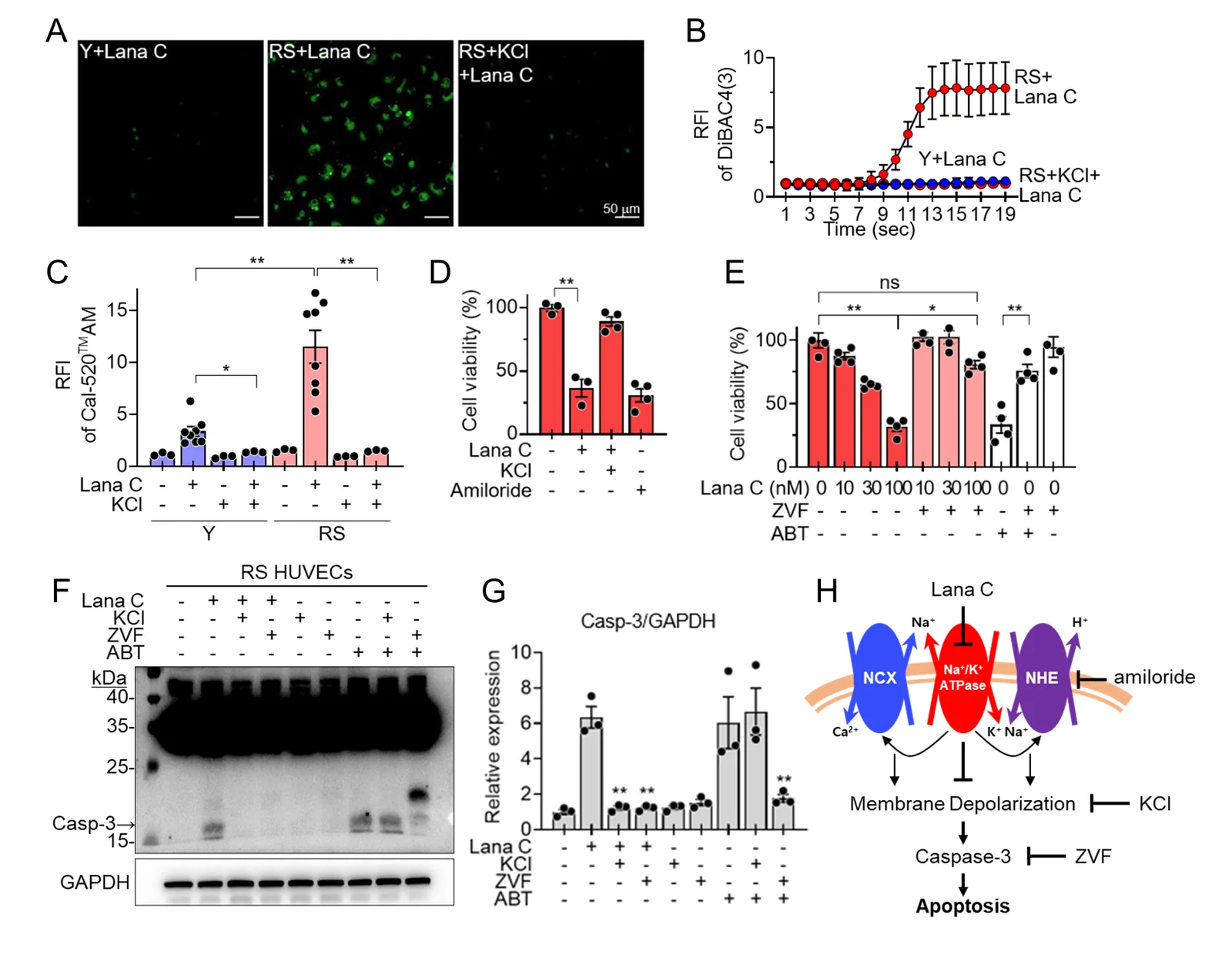
**Senolytic mechanism of Lana C in RS HUVECs.** Young or RS HUVECs were pretreated with fluorescent probe and KCl, and then Lana C treated (**A-C**). **(A)** DiBAC4(3) fluorescent microscopy. **(B)** DiBAC4(3) fluorescence intensity for plasma membrane potential in young or RS HUVECs (n=3 in each group). **(C)** Cal-520® AM fluorescence intensity for intracellular Ca^2+^ in young or RS HUVECs (n=3 or 8 in each group). RS HUVECs were pretreated with KCl (10 mM), amiloride (100 μM), ZVF (20 μM), and ABT-263 (100 nM) and then treated with Lana C (**D-G**). **(D-E)** Cell viability measured by cell counting (n=3 or 4 in each group). **(F)** Western blotting of active caspase-3 protein. **G.** Quantification of the levels of active caspase-3 protein (n=3 in each group). **(H)** Schematic illustration of Lana C’s mechanism of action. Values are presented as means ± SEM and data were analyzed with one-way ANOVA followed by a post-hoc test. *p<0.05, **p<0.01. Abbreviations: Y, young; RS, replicatively senescent; ABT, ABT-263; ZVF, Z-VAD-FMK; HUVEC, human umbilical vein endothelial cell; DiBAC4(3), bis-(1,3-dibutylbarbituric acid) trimethine oxonol; KCl, potassium chloride.

Cardiac glycosides are inhibitors of Na^+^/K^+^-ATPase and exert senolytic effects by increasing intracellular Na^+^ concentration, resulting in the induction of apoptosis by increasing intracellular Ca^2+^ and H^+^ levels via both the inhibition of the Na^+^/Ca^2+^ exchanger and the Na^+^/H^+^ exchanger [20]. Thus, we investigated whether Lana C also mediates senolytic activity through the inhibition of Na^+^/K^+^-ATPase. We measured the plasma membrane potential with specific fluorescence probes and intracellular Ca^2+^ and H^+^ concentrations. We used the DiBAC4(3) probe for plasma membrane potentials, Cal-520® AM for Ca^2+^, and pHrodo for H^+^, in young and senescent HUVECs treated with Lana C. The Lana C treatment showed a higher fluorescence intensity of DiBAC4(3) in senescent HUVECs than in young cells, suggesting the selective depolarizing effect of Lana C on the SCs (Figs. 5A and B, and Supplementary movie 1). When pretreated with KCl to restore the normal polarization of the plasma membrane, the fluorescence intensity of DiBAC4(3) enhanced in the SCs by the Lana C treatment was reduced (Figs. 5A and B, and Supplementary movie 1). The Lana C treatment induced a selective deleterious increase in intracellular Ca^2+^ in SCs, which was confirmed by a significantly higher fluorescence intensity of Cal-520® AM in the senescent HUVECs than in the young cells (Fig. 5C and Supplementary Fig. 2A). An increase in the intracellular Ca^2+^ in the SCs due to the Lana C treatment was repressed by KCl (Fig. 5C and Supplementary Fig. 2A). In addition, the Lana C treatment significantly increased the fluorescence intensity of the pHrodo dye in senescent HUVECs compared with young cells, indicating that Lana C induces selective detrimental acidification in SCs (Supplementary Fig. 2B). To further evaluate whether the selective depolarizing effects of Lana C on SCs could be involved in the senolytic activity of Lana C, we pretreated SCs with KCl and measured the senolytic activity. As expected, the senolytic activity of Lana C was abolished by the pretreatment with KCl (Fig. 5D). To confirm that the deleterious acidifying effect of Lana C in the SCs also contributed to its senolytic activity, we treated the SCs with amiloride, an inhibitor of the Na^+^/H^+^ exchanger that regulates intracellular pH [27, 28] and found that amiloride showed senolytic activity similar to Lana C (Fig. 5D).

#### Effects of Lana C on the apoptotic pathway

Furthermore, we investigated whether the senolytic activity of Lana C was mediated through the apoptotic pathway induced by the selective depolarization of the plasma membrane. Pretreatment with KCl reduced the expression of active caspase-3 proteins increased by Lana C (Fig. 5F). We also compared the senolytic activity of Lana C with ABT-263, which induces senescent cell-selective apoptosis [29]. Treatment of the RS HUVECs with Lana C increased the levels of the active caspase-3 protein and the senolytic activity of Lana C was inhibited by pretreatment with ZVF, a pan-caspase inhibitor [30], suggesting that apoptotic cell death is critical for mediating the senolytic activity of Lana C. Although the senolytic activity of Lana C was lower than that of ABT-263, the apoptotic pathway might be commonly involved in their senolytic activities (Fig. 5E-G). These results suggest that the senolytic activity of Lana C might be mediated through the apoptotic pathway induced by the selective depolarization of the plasma membrane and detrimental acidification (Fig. 5H and Supplementary Fig. 2B).

## DISCUSSION

Senescence is an essential mechanism in the maintenance of tissue homeostasis and protection against cancer. However, the accumulation of SCs over time is believed to contribute to tissue dysfunction, chronic inflammation, and the overall aging process. It plays a pivotal role in various age-related diseases, including cardiovascular diseases, metabolic disorders, and fibrosis [31-40]. Senotherapeutics, a novel class of therapeutic interventions aimed at modulating cellular senescence, have received considerable attention in recent years due to their potential to promote healthy aging and attenuating age-associated pathologies [41-44]. Among the most studied senotherapeutic interventions are senolytics such as dasatinib and quercetin [10], which are compounds that selectively induce apoptosis in SCs, and senomorphics including rapamycin and metformin [45], which alter the SASP and its deleterious effects. Promising preclinical results have demonstrated the rejuvenation of tissues and the extension of healthspan in animal models following senotherapeutic treatments [46]. However, senolytics are still in the early stages of development, and their long-term safety and efficacy in humans are yet to be fully established.

Cardiac glycosides have emerged as intriguing candidates in the search for effective senolytic agents [20]. Originally characterized as a cardiac glycoside used clinically for the treatment of cardiovascular diseases, digoxin has been recently investigated for its potential senolytic properties. This compound's dual pharmacological profile as an inhibitor of the transmembrane protein Na^+^/K^+^-ATPase and a regulator of cellular ion gradients makes it an attractive candidate for targeting and sensitizing SCs [20]. In this study, similar results were obtained using endothelial cells, and it was shown that the difference in ion concentrations inside and outside the aged cells due to Lana C was greater than that in young cells, indicating a senolytic effect.

In this study, we investigated the senolytic effects of Lana C on *in vitro* cellular senescence as well as *in vivo* age-related diseases in animal models at much lower concentrations as compared to earlier studies [20]. Lana C induces the apoptosis of senescent HUVECs and its senolytic activity is mediated through the plasma membrane depolarization induced by the inhibition of Na^+^/K^+^-ATPase. This is consistent with the action of other cardiac glycosides seen in earlier studies [20].

Senescent endothelial cells exhibit impaired function, including a reduced ability to regulate blood flow, maintain vascular tone, and modulate inflammation [47]. Endothelial dysfunction contributes to the loss of the protective barrier that the endothelium normally provides against plaque development [48]. We confirmed the inhibitory effect of Lana C on atherosclerotic plaque formation in *ApoE^-/-^* and *Ldlr^-/-^* mice, which are well-known animal models of atherosclerosis. In this study, *ApoE^-/-^* mice were fed HFDs for 12 weeks and administered Lana C from the 6^th^ week of the HFD, whereas *Ldlr^-/-^* mice were fed HFDs for 6 or 8 weeks, then changed to normal chow diets and treated with Lana C. Therefore, we attempted to determine the preventive effect of Lana C on atherosclerotic lesion progression in *ApoE^-/-^* mice as well as its therapeutic effect on atherosclerotic lesions in *Ldlr^-/-^* mice. The results showed that Lana C inhibited not only atherosclerotic lesion progression but also the initiation of atherosclerosis. Thus, the senolytic activity of Lana C could be beneficial for the prevention and treatment of atherosclerotic lesions in animal models.

Our findings presented in this study shed light on the senolytic potential of Lana C and its role as a novel therapeutic approach for age-related diseases. Understanding the mechanisms underlying its senolytic effects will not only contribute to our knowledge of cellular senescence, but also pave the way for the development of targeted therapies to enhance healthy aging and ameliorate age-related disorders.

However, we must acknowledge the limitations of this study, which focused primarily on molecular mechanisms. More extensive clinical investigations are needed to confirm the connections observed in the human population. Additionally, elucidating the precise molecular mechanisms and cellular interactions underlying the crosstalk between endothelial cells and other cells such as vascular smooth muscle cells remain an area of ongoing research.

## Supplementary Materials

The Supplementary data can be found online at: www.aginganddisease.org/EN/10.14336/AD.2024.1219.

## Data Availability

The data that support the findings of this study are available from the corresponding author upon reasonable request.
