# Supplementary material for: Lanatoside C, a Novel Senolytic, Ameliorates Atherosclerosis in Mice
Source: Aging Dis. 2025 Apr 21;17(3):1603–16. doi: 10.14336/AD.2025.1219 (PMC13128220; doi:10.14336/AD.2025.1219)
Supplement: Supplementary file 1 — The Supplementary data can be found online at: www.aginganddisease.org/EN/10.14336/AD.2024.1219. [file AD-17-3-1603-s.pdf]

SUPPLEMENTARY DATA

Lanatoside C, a Novel Senolytic, Ameliorates Atherosclerosis in Mice

Eok-Cheon Kim, Youlim Son, Seon-Hui Kim, Soo-Ji Kim, So-Young Park, Jae-Ryong Kim

© 2025. Kim EC et al. Published online at http://www.aginganddisease.org/EN/10.14336/AD.2024.1219

SUPPLEMENTARY DATA

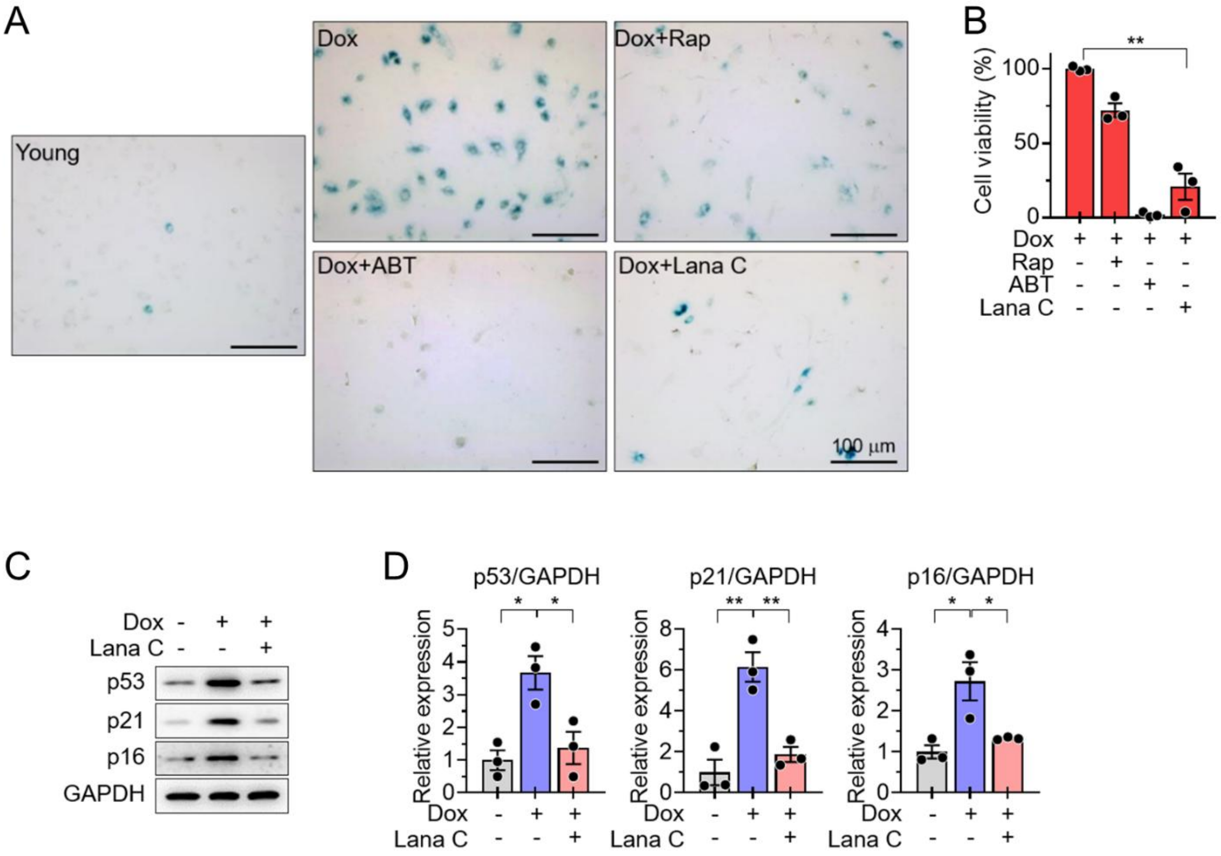

Supplementary Figure 1. Senolytic activity of Lana C in prematurely senescent HUVECs. Prematurely senescent (PS) HUVECs were treated with rapamycin (100 nM), ABT-263 (100 nM), and Lana C (100 nM) for 4 days and then analyzed the SAβG activity and cell viability. **A**. Representative SAβG staining images of young and PS HUVECs. **B**. Cell viability of PS HUVECs measured by cell counting (n=3 in each group). **C**. Representative Western blotting images. **D**. Quantification of the levels of p53, $p21^{Cip1}$, and $p16^{Ink4a}$ proteins normalized with GAPDH (n=3 in each group). Values are presented as means ± SEM and data were analyzed with one-way ANOVA followed by a post-hoc test. **p*<0.05. ***p*<0.01. Abbreviations: Lana C, lanatoside C; Dox, doxorubicin; Rap, rapamycin; ABT, ABT-263; HUVEC, human umbilical vein endothelial cell.

© 2025. Kim EC et al. Published online at http://www.aginganddisease.org/EN/10.14336/AD.2024.1219

SUPPLEMENTARY DATA

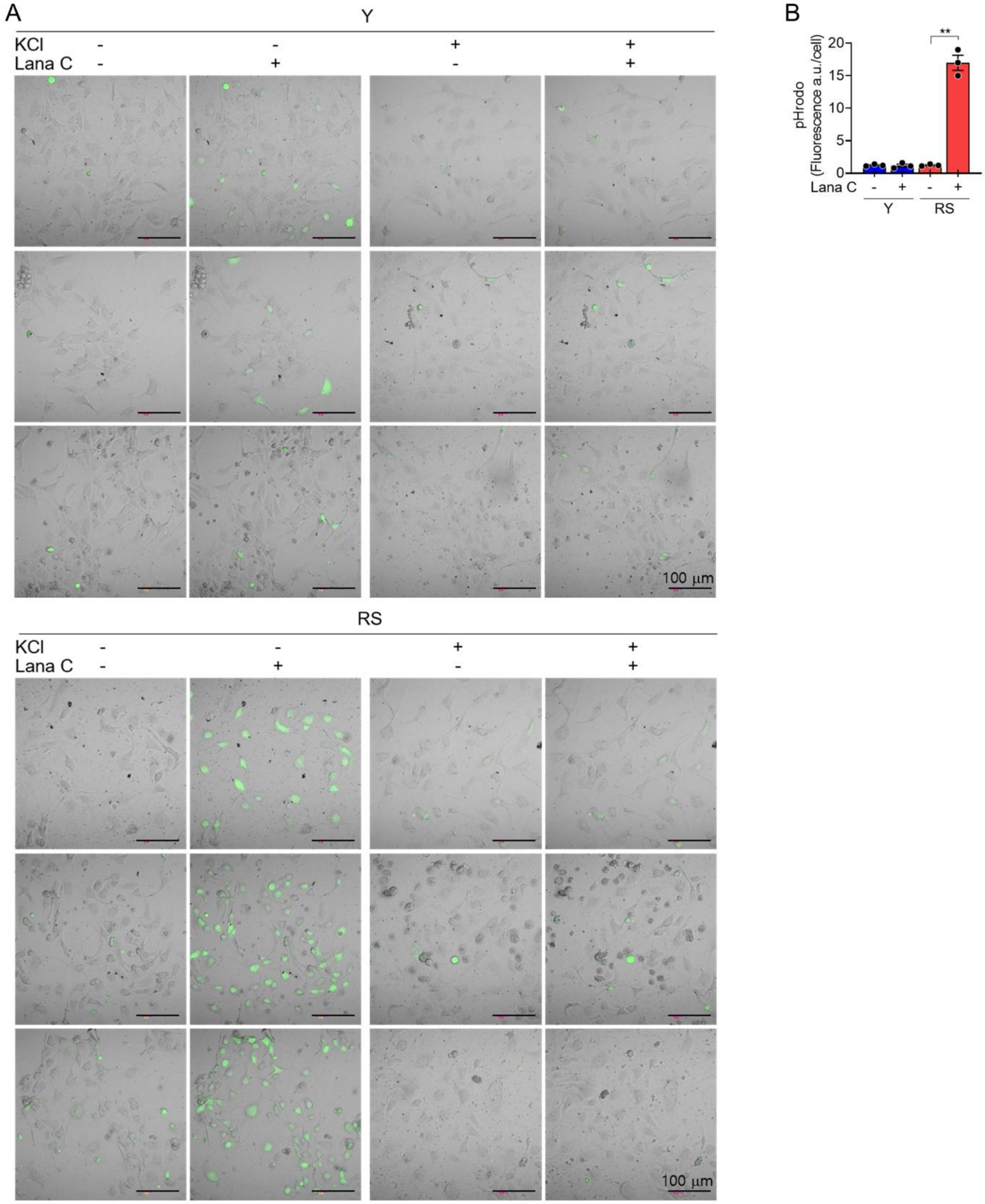

Supplementary Figure 2. The effects of Lana C on Ca^{2+} internalization and acidification. Young and RS HUVECs were pretreated with fluorescent probe and KCl (10 mM), then Lana C (100 nM) treated. **A**. Representative Cal-520® AM fluorescence images from 3 independent experiments of Y and RS HUVECs. **B**. pHrodo fluorescence intensity for intracellular pH in young or RS HUVECs (n=3 in each group). Values are presented as means ± SEM and data were analyzed with one-way ANOVA followed by a post-hoc test. ***p*<0.01.
Abbreviations: Lana C, lanatoside C; Y, young; RS, replicatively senescent.

© 2025. Kim EC et al. Published online at http://www.aginganddisease.org/EN/10.14336/AD.2024.1219

SUPPLEMENTARY DATA

Supplementary Table 1. Primary antibodies used in the study.

Protein	Company	Catalog number	Western blot, dilution
p53	Santa Cruz Biotechnology	sc-126	1:1000
$p21^{Cip1}$		sc-817	1:1000
$p16^{Ink4a}$		sc-377412	1:1000
Caspase-3	Cell Signaling	#9662S	1:1000
GAPDH		#5174S	1:3000
CD9	Abcam	ab92726	1:1000
Actin	Gene Tex	GT5512	1:3000

Supplementary Table 2. *ApoE*$^{-/-}$and *Ldlr*$^{-/-}$ mice used in the study.

Mouse genotype	Sex	Treatment	Age (Weeks)	Number
ApoE$^{-/-}$	Male	Vehicle (PBS)	10	9
			16	4
			30	4
		Lanatoside C	10	9
			16	5
			30	5
	Female	Vehicle (PBS)	10	7
		Lanatoside C	10	8
Ldlr$^{-/-}$	Male	Vehicle (PBS)	10	6
		Lanatoside C	10	8
	Female	Vehicle (PBS)	10	9
		Lanatoside C	10	9

Supplementary movie 1.

Measurement of cell membrane potential changes in young and RS HUVECs. Cells were pretreated with 10 mM KCl for 20 min, and then treated with Lana C. Abbreviations: Y, young; RS, replicatively senescent; HUVEC, human umbilical vein endothelial cell; KCl, potassium chloride.

© 2025. Kim EC et al. Published online at http://www.aginganddisease.org/EN/10.14336/AD.2024.1219
